# A Web—Based Respondent Driven Sampling Pilot Targeting Young People at Risk for *Chlamydia Trachomatis* in Social and Sexual Networks with Testing: A Use Evaluation

**DOI:** 10.3390/ijerph120809889

**Published:** 2015-08-20

**Authors:** Kevin Theunissen, Christian Hoebe, Gerjo Kok, Rik Crutzen, Chakib Kara-Zaïtri, Nanne de Vries, Jan van Bergen, Robert Hamilton, Marianne van der Sande, Nicole Dukers-Muijrers

**Affiliations:** 1 Department of Sexual Health, Infectious Diseases and Environmental Health, South Limburg Public Health Services, 6160 HA, Geleen, The Netherlands; E-Mail: christian.hoebe@ggdzl.nl; 2 Department of Medical Microbiology, School for Public Health and Primary Care (CAPHRI), Maastricht University Medical Centre (MUMC+), 6202 AZ Maastricht, The Netherlands; 3 Department of Work & Social Psychology, Maastricht University, 6200 MD Maastricht, The Netherlands; E-Mail: g.kok@maastrichtuniversity.nl; 4 Department of Health Promotion, School for Public Health and Primary Care (CAPHRI), Maastricht University Medical Centre (MUMC), Maastricht 6202 AZ, The Netherlands; E-Mails: rik.crutzen@maastrichtuniversity.nl (R.C.); n.devries@maastrichtuniversity.nl (N.V.); 5 Faculty of Engineering and Informatics. University of Bradford, Bradford BD7 1DP, UK; E-Mail: c.karazaitri@bradford.ac.uk; 6 Centre for Infectious Disease Control, RIVM National Institute of Public Health and the Environment, Bilthoven 3720 BA, The Netherlands; E-Mails: jan.van.Bergen@rivm.nl (J.B.); marianne.van.der.sande@rivm.nl (M.S.); 7 STI AIDS Netherlands, Amsterdam 1016 GB, The Netherlands; 8 Department of General Practice, AMC-University of Amsterdam, Amsterdam 1105 AZ, The Netherlands; 9 In-Fact, Bradford BD17 7DB, UK; E-Mail: bob@in-fact.com; 10 Julius Centre for Health Sciences and Primary Care, University Medical Centre Utrecht, Utrecht 3508 GA, The Netherlands

**Keywords:** *Chlamydia trachomatis*, web-based respondent driven sampling, peer-referral, social networks, sexual networks, partner notification, home-based test kits

## Abstract

*Background:* With the aim of targeting high-risk hidden heterosexual young people for *Chlamydia trachomatis* (CT) testing, an innovative web-based screening strategy using Respondent Driven Sampling (RDS) and home-based CT testing, was developed, piloted and evaluated. *Methods:* Two STI clinic nurses encouraged 37 CT positive heterosexual young people (aged 16–25 years), called index clients, to recruit peers from their social and sexual networks using the web-based screening strategy. Eligible peers (young, living in the study area) could request a home-based CT test and recruit other peers. *Results:* Twelve (40%) index clients recruited 35 peers. Two of these peers recruited other peers (n = 7). In total, 35 recruited peers were eligible for participation; ten of them (29%) requested a test and eight tested. Seven tested for the first time and one (13%) was positive. Most peers were female friends (80%). Nurses were positive about using the strategy. *Conclusions:* The screening strategy is feasible for targeting the hidden social network. However, uptake among men and recruitment of sex-partners is low and RDS stopped early. Future studies are needed to explore the sustainability, cost-effectiveness, and impact of strategies that target people at risk who are not effectively reached by regular health care.

## 1. Background

Screening of (a)symptomatic patients is considered an important strategy for controlling the transmission of *Chlamydia trachomatis* (CT) among young people [1]. Early detection and treatment prevents the infection from spreading and causing further morbidity. Most CT infections are found among young heterosexual people [1,2,3]. 

The number of reported cases of CT has increased worldwide in the past years [1,2,3]. With approximately 100 million new cases of CT every year, many CT infections still remain hidden to health care [3]. Approximately 30,000 infections are diagnosed in Sexually Transmitted Infections (STI) clinics and General Practitioner (GP) practices amongst young people each year in The Netherlands [2]. However, there are an estimated 60,000 new CT infections among young people [4] suggesting that half of the CT infections remain hidden, *i.e*., invisible to health care, in The Netherlands. Many young people at risk for CT may not seek sexual health care [3] due to the asymptomatic nature of the bacterial infection and other barriers such as fear for the sampling methods, a positive test result, and associated stigmatization and privacy concerns [1,5,6,7,8].

Several attempts have been made nationally and internationally, beyond regular sexual health care, to increase CT testing among young people at risk for CT. Initiatives in The Netherlands, United States, Canada, Sweden, Norway and the United Kingdom focused on population-based screening using the Internet and home-based testing to engage young people in CT testing [9,10,11,12,13,14]. Although such screening methods are appreciated amongst their users [13,15], both participation and CT positivity rates in such population-based initiatives are generally low [9,10,11,12,16]. Furthermore, this method is not found to be cost-effective in The Netherlands [17].

Other approaches have been developed with the aim to reach out to the hidden CT positives by specifically targeting sexual partners of young CT positive people. Such partner notification approaches use a combined web-based system and/or home-based testing, building on the understanding that partner-notification is essential in the management of CT [18,19,20,21,22]. This is because timely notification, testing and treating of partners prevents transmission and (re)-infections. Web-based partner notification is complementary to standard face-to-face or telephone notification by providers (provider referral) and/or patients (patient referral) and can be used to send personal or anonymous emails, e-cards or mobile text messages (SMS) to motivate sexual partners to test for CT [6,19,20,22]. Web-based partner notification is well accepted among its users to notify others, but information about its actual use and its outcomes is scarce, especially with respect to the proportion of partners actually seeking testing and care [6,20,23]. A comparison was made between test uptake among partners by using home-based test kits compared to the regular testing by a health care provider in a randomized controlled trial. Results showed that more partners were tested and found to be CT positive with home-based test kits [21].

Other approaches [24,25] have explored the use of social networks to access hidden high-risk young people for CT testing. These approaches use peer-led testing whereby young people recruit their social networks to test for CT using home-based test kits. Peers influence each other’s attitudes and actions with respect to sexual behaviour and STI testing [26]. In reality therefore, not only sexual partners but also friends, *i.e*. the social network, of a CT positive person may be at higher risk for CT, although they do not share a sexual relationship [6,26]. Interestingly, approaches based on CT testing using social networks suffered from poor test kit uptake. Additionally, they have not explored using CT positives as recruiters of social network members [24,25]. 

Respondent driven sampling (RDS) is another method that has been used for many years, with some success, to reach out a targeted hidden population [27]. RDS initiates the search by using index clients (e.g., a person with an infection) to recruit and motivate network members to test (first wave). These members can motivate their own network members to get tested (second wave) and so on [27]. RDS has been demonstrated successful in finding new cases through social network members (non-sexual relationships) in studies with HIV [28] and syphilis [29]; cases who otherwise would not be reached when only sexual partners had been targeted (*i.e*., partner notification). Limited human and economic resources led to Internet based methodologies for conducting RDS in networks, which have shown effective in several studies. Until now, web-based RDS has been applied to infectious diseases [30] and drug and alcohol use [31] with some success. We hypothesize that web-based RDS may also be a potential novel strategy to reach young people at risk for CT who are not reached by regular care efforts, using their sexual and social networks and home-based test kits.

To test this hypothesis, we developed a novel web-based RDS outreach screening strategy, using a systematic approach, *i.e*., the Intervention Mapping (IM) protocol [32]. The strategy combines recruitment of the social and sexual network members starting with CT positive young people, and home-based CT testing. We piloted this strategy to test and evaluate its use (uptake and usability). Such evaluations have rarely been described for most other similar projects, yet are hoped to provide the necessary learning points for future CT control strategies that target social and sexual networks of high-risk young people, in particular those not reached by existing regular care.

## 2. Method

### 2.1. Setting and Intervention Development

The intervention was piloted at the STI clinic of the Public Health Service South Limburg, The Netherlands. This clinic provides about 6500 STI consultations annually, offering free examination and treatment. The development of the intervention has been described in detail in Theunissen *et al.* [32]. In short, the intervention program components and specific materials were determined, based on existing theories and evidence, using the Intervention Mapping (IM) protocol [33].

### 2.2. Intervention Pilot Implementation

**Figure 1:**
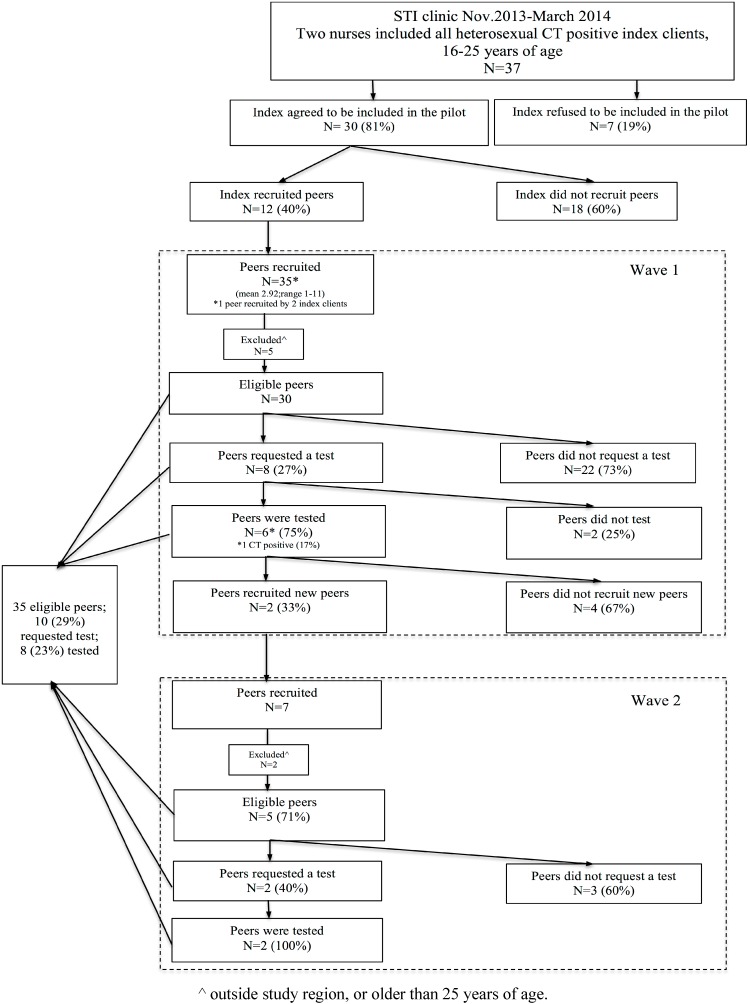
Flowchart of the selection of the study population and recruitment of their peers.

From November 2013 to March 2014, two sexual health care nurses piloted the intervention in their daily practice among CT positive young people and their social and sexual network members. Initially, the intervention was developed to consist of two interfaces: (1) a public website (online interface) for young people to recruit peers (*i.e*., https://www.SafeFriend.nl) and (2) a secure private interface which enables nurses to invite, motivate and support index clients to start a recruitment-chain during the treatment consultation at the STI clinic. Index clients were CT positive young people who were aged between 16 and 25 years, lived in South Limburg and visited the STI clinic for CT treatment. Young men who reported having sex with men (MSM) were excluded from participation, because according to national guidelines they are recommended to serologically test for other STI as well (*i.e*., HIV). The nurses asked all eligible index clients during the treatment consultation to participate and recruit anonymous or non-anonymous their sexual and/or social network members (peers) via the web-based screening strategy using email. They could login at the website (https://www.safefriend.nl) with their personalized credentials. The recruited peers (first wave) were then able to login and A) request a home-based urogenital CT test kit for themselves and B) recruit their social and sexual network members. The newly recruited peers then followed the same procedures, and so on; with a maximum of five waves. Peers in the pilot were eligible if they were aged 16–25 years; they were living in South Limburg and were not a man reporting having sex with a man. Excluded peers were advised to visit their GP. Home-based test kits could be requested to be sent to home addresses or collected at the STI clinic and were then mailed by the peers to the laboratory. Index clients and peers who did not finish the online process of the screening strategy or who did not return a home-based test kit received an email reminder two weeks after the first login. The nurse informed all CT positive peers by telephone and recommended treatment at the STI clinic South Limburg. The selection of the study population and the recruitment of peers is depicted graphically in Figure 1.

### 2.3. Pilot Evaluation

The evaluation study was approved by the Ethics Committee of the Psychology Department of Maastricht University (ECP-13-4-054) prior to implementation*.* The evaluation comprised five steps of the intervention process: (1) technical development, (2) inclusion of index patients by nurses, (3) participation of index clients, (4) participation by peers, and (5) the treatment of CT positives.

(1) Technical development

To evaluate the technical part of the screening strategy, the functionalities used during the pilot were compared to those described in the IM protocol (which had been written before the pilot) [32]. The following functionalities were evaluated: the interfaces for nurses and young people, request of home-based test kits, recruitment process via email and SMS, and anonymous and non-anonymous recruitment.

(2) Inclusion of index patients by nurses

The percentage of index clients included was calculated and basic characteristics (age, sex, education level and number of sex partners during the last six months) were compared between included and not included clients using chi-square analyses. The number of clients who refused to participate, and reasons for refusal were analysed from a structured paper questionnaire which the two nurses filled in for eligible index clients. This questionnaire was used by the nurses for the partner notification and to invite index clients for the pilot. Information registered included the number of sexual partners in the past six months, partners already notified, and reasons for non-participation. Also the additional time needed to explain the pilot was included to estimate the workload for providers. To evaluate the providers’ acceptability and workload, two weekly appointments of one hour were held with a researcher during the time of the pilot. During these appointments the researcher systematically recorded all comments (including positive and negative experiences) of the providers with the screening strategy and index clients.

(3) Participation of index clients

To evaluate index participation, *i.e*. recruitment, we used process observations (coded online collected data *i.e*., login dates, email addresses, birth dates, postcode area, sexual behaviour, actual recruitment, type of message sent, and type of relation between participants). The participation rate was calculated as the ratio of index clients who actually participated (recruiting peers from their social and/or sexual networks) by the number of index clients who were included by the two nurses in the pilot. Basic characteristics (age, sex and type of relationship with index, education level and number of sex partners last six months and test history) of participants and non-participants were compared using chi-square analyses.

(4) Participation by peers

Peer participation data, *i.e*. recruitment of network members, the type of relationships (*i.e*., best friend, friend, acquaintance, steady partner, casual partner, steady casual partner) and the number of waves reached, were retrieved from the process data and the PN questionnaires.

(5) The treatment of CT positives

The proportion of diagnosed CT was based on the biological assessment of the urogenital home-based self-taken samples (urine for men and vaginal swab for women) using a nucleic acid amplification test (NAAT: PCR, Cobas 4800, Roche, Pleasanton, CA, USA). The treatment status of positives was assessed by reviewing the medical records for tested cases.

## 3. Results

### 3.1. Technical Development

The nurse interface, to invite index clients at the treatment consultation for our website, could not be used. Therefore nurses could not start the recruitment of peers by index clients at the STI clinic. Instead, the nurses sent the index clients an email containing a personal link to the website to use the online facility at home to recruit their peers.

Further, there were technical problems with the online interface for young people whereby only email as a method for participation could be used. The originally designed text messaging function was lacking. Moreover, in the first two weeks of the pilot six participants could not use their personal links to login (technical error). These participants were sent a second personal link. Finally, the website was offline during two weeks (server problems). In spite of the technical problems several technical features designed to increase usability were included in the strategy, *i.e*., providing the option to request home-based test kits and to recruit peers anonymous or non-anonymous.

### 3.2. Inclusion of Index Clients

During five months two female nurses served 37 eligible index clients who were all invited to participate and who filled in the structured paper partner notification questionnaire. The additional time that nurses needed for this inclusion and registration was between five to 15 minutes per index. Index clients were mostly women, between 21 and 25 years of age, highly educated and had had no more than two sex partners in the past six months (Table 1).

**Table 1:** Characteristics of index clients who recruited peers and non-recruiters.

				Index Included	
	All Eligible Index Clients (n = 37)	Eligible Index Clients Refused (n = 7) *	Eligible Index Clients Included (n = 30) *	Index Clients Non-Recruiter (n = 18) *	Index Clients Recruiter (n = 12) *
	% (n)	% (n)	% (n)	% (n)	% (n)
*Age (years)*					
17–20 years	43.2 (16)	71.4 (5)	36.7 (11)	38.9 (7)	33.3 (4)
21–25 years	56.8 (21)	28.6 (2)	63.3 (19)	61.1 (11)	66.7 (8)
*Sex*					
Female	83.8 (31)	100.0 (7)	80.0 (24)	77.8 (14)	91.7 (11)
Male	16.2 (6)	0.0 (0)	20.0 (6)	22.2 (4)	8.3 (1)
*Education level ^*					
University and Higher Professional Education	68.6 (24)	57.1 (4)	71.4 (20)	62.5 (10)	83.3 (10)
Intermediate Vocational Education	31.4 (11)	42.9 (3)	28.6 (8)	37.5 (6)	16.7 (2)
*Number of sex-partners past six months*								
1–2	64.9 (24)	85.7 (6)	60.0 (18)	61.1 (11)	58.3 (7)
3 or more	35.1 (13)	14.3 (1)	40.0 (12)	39.9 (7)	41.7 (5)

^ 2 missing, * non-significant.

Eventually, 30 of 37 eligible index clients agreed to be included in the pilot; thereby the intended uptake was 81%. They all received an invitation email with a personal link to login on the web-based intervention at home and recruit peers to test for CT. No statistically significant differences (all *p* > 0.2) were observed between clients who refused to be included (n = 7; 19%) and index clients who accepted to be included (Table 1). Reasons reported by eligible index clients to not be included in the pilot were shame, the unavailability of an email address of a sex partner, a sex partner had already been notified and friends were in a steady relationship and/or already tested. The seven index clients who refused to be included reported a total of 19 sex partners and said that they already had notified nine of them.

During the weekly evaluation sessions, the nurses stated that the structured partner notification questionnaire, that was used in this pilot, helped them to systematically discuss the notification of sex partners with their clients. They felt also more motivated to encourage index clients to notify their sexual networks.

The nurses also said that they were more aware of the CT risk among social network members of CT positive clients and emphasized the importance of their recruitment. The web-based strategy also provided them with previously lacking and important feedback on the effectiveness of their work process (*i.e*., visual notification of sex partners and friends).

Nurses also noted shortcomings. The lack of an online nurse interface was a missed opportunity to provide support and to persuade index clients during their treatment consultation to use the web-based strategy and recruit peers. Index clients mentioned to the nurses that they preferred receiving and sending text messages instead of email.

### 3.3. Index Participation: Recruitment of Peers 

Of the 30 index clients who received a personal link to login on the website 18 persons did not recruit their peers (Table 1). The actual uptake was therefore 40% with 12 index clients participating by recruiting peers. No statistically significant differences (all *p* > 0.2) were found between index clients who did not recruit peers and those who did (Table 1).

**Figure 2:**
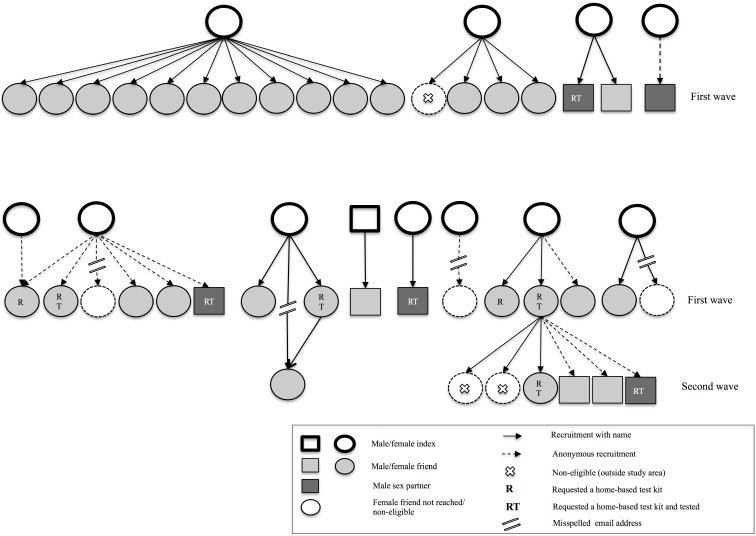
Recruitment among index clients and first wave peers.

In total, the 12 participating index clients recruited four sexual (two anonymous) and 31 social (only six anonymous) network members during the first wave (Figure 2). Of the sexual partners two were casual male partners and two were steady partners (one male, one female). The 31 social network members included four best friends and 27 friends; 29 were women. The 12 index clients that recruited their peers reported in total 29 contactable sex partners of whom one was already tested and 15 (52%) had been notified. Of the 14 sex partners (48%) that were not yet notified, four (29%) were recruited via our screening strategy In total this resulted in only 9%, of all sex partners mentioned by the 30 included index clients, that were reached by our screening strategy.

### 3.4. Peer Participation: Requesting a CT Test and Recruitment of Other Peers

Of the 35 recruited peers, four email addresses of social network members were misspelled; these peers were therefore not reached by the intervention. The 31 effectively recruited peers were offered to login to the website to request a home-based CT test kit and also recruit their peers. One social network member was not eligible to participate in the pilot. Of the 30 eligible recruited first wave peers most (n = 28) did not recruit their peers. The remaining two eligible first wave peers (female friends) logged in and eventually recruited one steady male sex partner (anonymous) and six friends (two anonymous; two men). Two recruited friends were not eligible to participate. None of the five eligible peers in the second wave recruited new peers (Figure 2). 

Of all (n = 14) recruited peer networks in the first and second wave, 43% (n = 6) included three or more persons. Of the 30 eligible first wave peers 27% (n = 8) requested a home-based test kit. Most of these peers were female, and never tested before (Table 2). The test kit return rate was 75% (n = 6; n = 3 female friends, n = 3 male sex partners). One (13%) peer (male sex partner) was found CT positive. Of the five eligible second wave peers 40% (n = 2) requested a test kit, one female friend and one male sexual partner who were both CT negative. 

Of all index clients who recruited peers (n = 12), 50% (n = 6) did so after they had received a reminder. Twenty-six reminders were sent to finish the online process (recruiting others or requesting a home test) to peers in the first wave. None of these peers who received a reminder recruited peers and only one female requested a home-based test kit. Six reminders to finish the online process were sent to peers from wave two. None of them encouraged peers or requested a test kit. 

**Table 2:** Characteristics of eligible peers.

	Eligible peers who did not request a test/nor recruited another peer) (n = 25)	Eligible peers who requested a home-based test but not recruited other peers (n = 8) *	Eligible peers who requested a home-based test and recruited other peers (n = 2) *
	% (n)	% (n)	% (n)
*Age (years)*			
17–20 years	N/A	50.0 (4)	0.0 (0)
21–25 years	N/A	50.0 (4)	100.0 (2)
*Sex and relationship with index*			
Female friends	80.0 (20)	50.0 (4)	100.0 (2)
Male Friends	16.0 (4)	0.0 (0)	0.0 (0)
Male sex partners	4.0 (1)	50.0 (4)	0.0 (0)
*Education level*			
University and Higher Professional Education	N/A	100.0 (8)	100.0 (2)
Intermediate Vocational Education	N/A	0.0 (0)	0.0 (0)
*Number of partners past six months ^*			
1–2	N/A	71.4 (5)	100.0 (2)
3 or more	N/A	28.6 (2)	0.0 (0)
*Test history*			
Tested before for CT	N/A	0.0 (0)	50.0 (1)
Never tested for CT	N/A	100.0 (8)	50.0 (1)

^ 1 missing, * non-significant.

### 3.5. Treatment of CT Positive Peers

One peer was CT positive. He visited the STI clinic for treatment, additional testing and routine partner notification. 

## 4. Discussion

We report the evaluation of the first CT outreach screening strategy that combined web-based social and sexual peer-referral (*i.e*., Respondent Driven Sampling) and home-based sampling to reach hidden high-risk young people for testing. We found several positive factors and some shortcomings that should be taken into account in future CT (re)-screening strategies for young people (Table 3). Positive factors included substantial recruitment of peers among index clients (40%) with a mean number of 3 peers recruited. Mainly non-sexual friends were recruited who would otherwise be missed in conventional partner notification. The strategy reached a hidden population as most tested peers (88%) were never tested before. In theory these friends are at higher risk, because their sexual behaviour is influenced by their friends and sex partners who are CT positives (index clients). However, this influence could neither be confirmed nor rejected by our study. Health care providers experienced more feedback on their daily work, were more aware of the CT risk in networks and were more motivated to encourage networks to recruit each other for CT testing. The strategy also provided information on the proportion of friends and sex partners actually seeking testing and care; valuable information that is scarce in current evaluation studies. The shortcomings included technical difficulties, peer-referral chains that were small, low number of sexual partners reached and few participating men.

**Table 3:** An evaluation of use of the web-based screening strategy; a summary of the positive factors and shortcomings.

	Positive factors	Shortcomings
Usability by health care providers	(2) Providers feel more motivated (compared to before the pilot) to encourage index clients to recruit their social and sexual networks	(1) Lack of an online interface for providers (this would allow to start recruitment during STI clinic consultation avoiding delay)
	(2) Structured partner notification process	
	(2) Feedback on partner notification result (which provides information on the proportion friends and sex partners actually seeking testing and care; to evaluate care effectiveness)	
	(1) Secure, fully automated system (5) Automated link with referral of CT positives to further health care and actual treatment and care	
Usability by young people	(3,4) Personal messages	(1) Online and login problems (technical)
	(4) Home-based test kits	(1) Misspelling of email addresses. No system (technical) feedback to recruiting peer of email error
	(3,4) System reminders	
		(1) Lack of availability of short message service (SMS) texts to recruit peers (this is likely to be the most used communication method)
Uptake by young people	(3,4) Recruitment of social network members (peer-referral)	(3,4) Low number of recruited waves
	(3,4) Moderate network sizes	(3,4) Few men reached
		(3,4) Few sex-partners reached
		(3) A number of indexes with intended uptake still did not actually participate

1–5 refers to the steps of the evaluation process (1) technical development, (2) inclusion of index patients by nurses, (3) participation of index clients, (4) participation by peers, (5) treatment of positives

Several positive factors were noted. It was encouraging to learn that the strategy was highly acceptable among the providers involved in the pilot. They stated that it helped them better structure their consultation, and that it increased their awareness about the CT risk among social network members of CT positive young people. Therefore, they felt more motivated to encourage and support index clients to recruit their network members. Also, they stated that the strategy provided them with important feedback on the effectiveness of their work process, feedback that so far had been lacking [34].

Another finding that we consider promising is the substantial recruitment of peers among index clients (40%) with a mean number of three peers recruited. Index clients and first wave peers almost exclusively recruited friends to test for CT. In conventional partner notification, such social network members are not targeted hence remain hidden to care. The strategy could potentially enhance current STI outreach work. This outreach work aims to increase testing in high-risk populations outside STI clinic walls. A comparison with other studies that use peer referral via Internet is difficult to make, because they have either focused on the recruitment of sex partners [19,20,22] or only studied the acceptability among its users [19,20]. Studies that have focused solely on the recruitment of non-sexual contacts (friends) did not use the Internet and CT positive young people to recruit peers [24,25].

The recruitment of young people is aimed at testing and treating them when needed, which is fundamental to decreasing the transmission. Home-based test kits were requested and returned by 23% of all eligible recruited peers (social and sexual). This return rate is somewhat higher compared to a Dutch population-based screening strategy, where 16% of the invited young people between 16 and 29 years old requested online a CT home-based test kit and returned it for testing [9]. However, more importantly to note is that our intervention reached young people who were hidden to care, as 88% had never been tested before and of whom 13% was CT positive (one sex partner). 

Shortcomings of the strategy included technical problems. Due to a lack of funding, the web-based screening strategy in the pilot was not fully developed, lacking some of the components that were originally designed to increase usability. This limited its effectiveness to some extent, as providers could not start a recruitment-chain at the STI clinic (where they could oversee and support actual peer-recruitment). Such combination of patient and provider referral could increase the notification of sex partner, as discussed in several studies [6]. Further, messages could only be sent by email instead of SMS, while the latter is frequently used by young people. In a study about web-based partner notification among STI clinic clients only 15% chose emails and 84% mobile text messages to notify their sexual partners of a possible STI infection [22]. Additionally, email addresses were misspelled. However, the same issue could occur with mobile phones. In both cases, it is very difficult to know if messages have been received or not and to inform the sender of the mistake. A possible solution may be to automatically retrieve mobile numbers of a person’s contacts, which however may be difficult to achieve in a secure web-facility.

Furthermore, even though recruitment by index clients was substantial, it was also selective in that mostly female social networks were reached. The aim of using the strategy to recruit sexual partners (as in web-based partner notification) is thereby clearly not reached as only few sex partners were recruited. Of all contactable sex partners, more than half of them had not yet been notified by the eligible index clients during the CT treatment consultation. Furthermore, only four index clients (13%) recruited a sex partner via our strategy. This low recruitment rate agrees with 14% recruitment of sex partners observed in another study, where STI positive clients received a personal code for online recruitment of their sex partners for testing [22]. Reasons for the poor recruitment of their sex partners as opposed to social networks could be the intimate history persons have had with sexual partners which can lead to anticipated negative reactions (*i.e*., blame and aggression towards or from partners) [7] and stigma surrounding disclosure and encouragement of others to test [34]. Overall, females were better represented in our study, which is in line with an earlier study [25] that showed that men were more reluctant to distribute kits to their peers and were more embarrassed to discuss sexual health-related issues. Women have been found more likely to recruit men in other web-based RDS approaches, addressing respiratory infections, as well [30].

Although, peer-referral combined with a web-based RDS method was judged highly promising and feasible during the development of the intervention [32], only two waves were created in this pilot study. We know that stigma plays an important role in the process of disclosure to and encouragement of friends and sexual partners [35,36,37]. In a study among CT tested and never tested young people between 16-24 years, participants used selective disclosure and encouragement as an effective strategy to manage stigma. They stated that they only disclosed their testing experience to a small and selective group that provides them with emotional support and empathy. They fear stigmatizing reactions from others [37]. To overcome this stigma the screening strategy already had embedded the option to notify friends and sex partners anonymous. Still, people did not often use this option. More than two third of all peers in this study were recruited with personal messages (40% among sex partners and 78% among friends). Factors other than stigma may be at play. For example, a web-RDS study addressing much less stigmatized infections (respiratory infections) in young people, also achieved only few waves [30]. Future interventions should explore how to reach high-risk network members outside the small networks. A possible method to combine with our screening strategy could be Motivational Interviewing (MI). MI is a client-centred approach for eliciting behaviour changes by helping people to feel confident to perform a particular behaviour according to their needs and abilities [38]. When applied in partner notification training for sexual health care providers MI has been shown to improve skills and behaviour in dealing with patients’ resistance towards PN [39]. This way, clients may also be more motivated to encourage peers outside their trusted sexual and social networks. Messages could be send to encourage index clients to remind their peers to mail their CT test to the laboratory. 

Some limitations of our study design need to be considered when interpreting our results. Firstly, the number of included nurses and eligible CT positive index clients was small. This may hamper generalizability of our results to respectively the broader population of STI clinic health care providers and CT positive young people. Secondly, only heterosexual young people at risk for CT were eligible to participate in the pilot. Therefore, it is unknown whether the results can be extrapolated to other target groups such as young MSM or other STIs (e.g., syphilis or HIV). Thirdly, the cost-effectiveness and sustainability of the screening strategy was not evaluated. However these factors will influence future implementation of a screening strategy in daily practice.

Other limitations concern the study findings. Firstly, non-participation of index clients and peers in semi-structured interviews was high. This limited the assessment of usability of the screening strategy developed and indicated how difficult it is to conduct interviews with this hard-to-reach group. Secondly, the assumption that recruited clients’ friends are at higher risk for CT (as they are in the same network as a CT positive person), the actual higher risk could neither be confirmed nor rejected by the data. None of the reached friends tested CT positive, friends included few lower educated persons and relatively few persons with a high number of partners. Further evaluation to assess the CT risk of reached social networks is therefore needed.

## 5. Conclusions

With pretty reasonable recruitment and test rates among respectively index clients and peers who have never been tested previously we feel that the intervention provided proof-of-concept and succeeded to reach young people who are currently not accessing CT testing. Most of the recruited peers were social contacts that would normally be missed through conventional partner notification processes. A positive impact of the strategy was experienced by nurses in daily STI care practice. However, it should be noted that only two RDS waves were created. Also, penetration in the sexual network was poor and it is unclear whether the social network accessed with testing did comprise high-risk persons. Other shortcomings included technical problems, which however could be resolved (*i.e*., using SMS instead of email). The use of peer-referral in combination with home-based test kits seems promising, but its combined use with online RDS should be studied and developed further to explore if more waves, sex partners and men can be reached. Learning points as identified here can help to inform health care strategies that aim to increase CT testing in high risk populations, especially those that employ social and sexual networks. Future studies are needed to also explore the sustainability and cost-effectiveness of these approaches.
